# Atypical Clinical Presentation of Crohn’s Disease as Diffuse Abdominal Lymphadenopathy and Venous Thrombosis: A Difficult to Diagnose Case

**DOI:** 10.7759/cureus.5805

**Published:** 2019-09-30

**Authors:** Ahmad Raza, Vincent Chan, Muhammad Arslan Cheema

**Affiliations:** 1 Internal Medicine, Abington Hospital-Jefferson Health, Abington, USA

**Keywords:** abdominal vein thrombosis, crohn's disease, lymphadenopathy

## Abstract

Crohn’s disease (CD) is an inflammatory bowel disease with clinical manifestations that are more variable than those of ulcerative colitis. It can manifest with a wide range of gastrointestinal as well as extra-intestinal symptoms and at times it becomes difficult to diagnose because of presenting variability. Here we present a case of a young male who presented with diffuse abdominal lymphadenopathy with abdominal vein thrombosis and found to have CD.

## Introduction

Crohn’s disease (CD) is a chronic relapsing inflammatory bowel disease (IBD). It is characterized by transmural granulomatous inflammation which can affect any part of the gastrointestinal tract, most commonly the ileum but can involve the colon or both. Variable clinical presentations of CD coupled with a lack of gold standard testing to diagnose the disease makes it a challenge for clinicians on a day to day basis. Its presentation can be as subtle as generalized fatigue [1] or chronic diarrhea, defined as a decrease in fecal consistency for more than four weeks [2]. Abdominal pain (70%), weight loss (60%), and bloody or mucoid stools (40%-50%) are also common findings in CD [3]. The most commonly observed extraintestinal manifestation in primary peripheral arthritis (33%), aphthous stomatitis, uveitis, erythema nodosum, and ankylosing spondylitis can be seen whilst pyoderma gangrenosum, psoriasis, and primary sclerosing cholangitis are relatively uncommon [4]. Venous thrombosis is also a well-known presentation and/or complication of CD [5-6]. Despite being a chronic inflammatory condition with nongranulomatous inflammation, diffuse lymphadenopathy with venous thrombosis remains a rare presentation of CD.

## Case presentation

We present a case of a 28-year-old African American male with the previous history of iron deficiency anemia who presented to us for the evaluation of abdominal pain, lower extremity swelling, and fatigue. His anemia was attributed to the previous infection with *Helicobacter pylori* (*H. pylori*). His symptoms were chronic for months except for the abdominal pain that started about a month ago. He was hemodynamically stable with exam findings of mild abdominal tenderness and 2+ bilateral pitting edema. On lab work, he was found to have severe microcytic anemia (hemoglobin 5.2 g/dL) and was transfused with packed red blood cells (PRBCs) for symptomatic anemia and additional lab work was sent. Peripheral blood smear reviewed by the hematologist was grossly abnormal with teardrop cells, poikilocytosis, and nucleated RBCs but without blasts or dysplastic neutrophils. His iron studies were normal. His abdominal ultrasound showed evidence of a soft tissue mass measuring 3.0 cm x 1.5 cm x 1.8 cm, seen within the portal confluence and extending into the splenic vein. There was also a mass versus complex collection along the anterior pancreatic body. Upper gastrointestinal (GI) endoscopy done for further evaluation showed prepyloric ulcers along with “hard fullness” in the gastric region suggestive of extrinsic mass without signs of inflammation in the small bowel wall.

**Figure 1:**
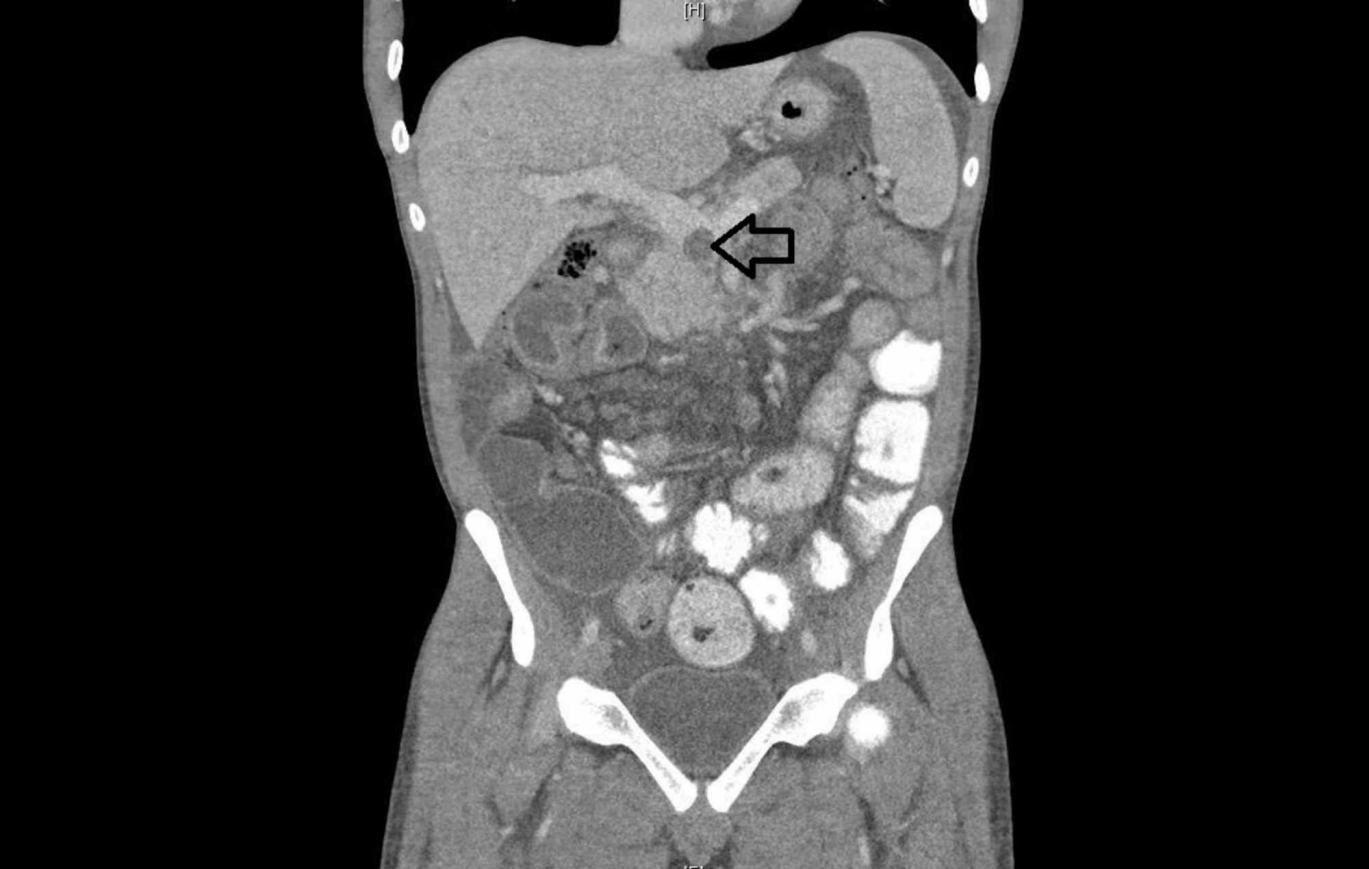
Sagittal view of the CT scan abdomen/pelvis showing mesenteric vein thrombosis (arrow) at the level of portal vein and splenic vein confluence.

Considering the above findings, a CT scan of the abdomen was done that showed multiple thrombi in the portal system. There was significant venous thrombosis at the level of the superior mesenteric vein (Figure 1). Venous thrombosis started from intrahepatic portal veins and extended to the mesenteric vein.

**Figure 2:**
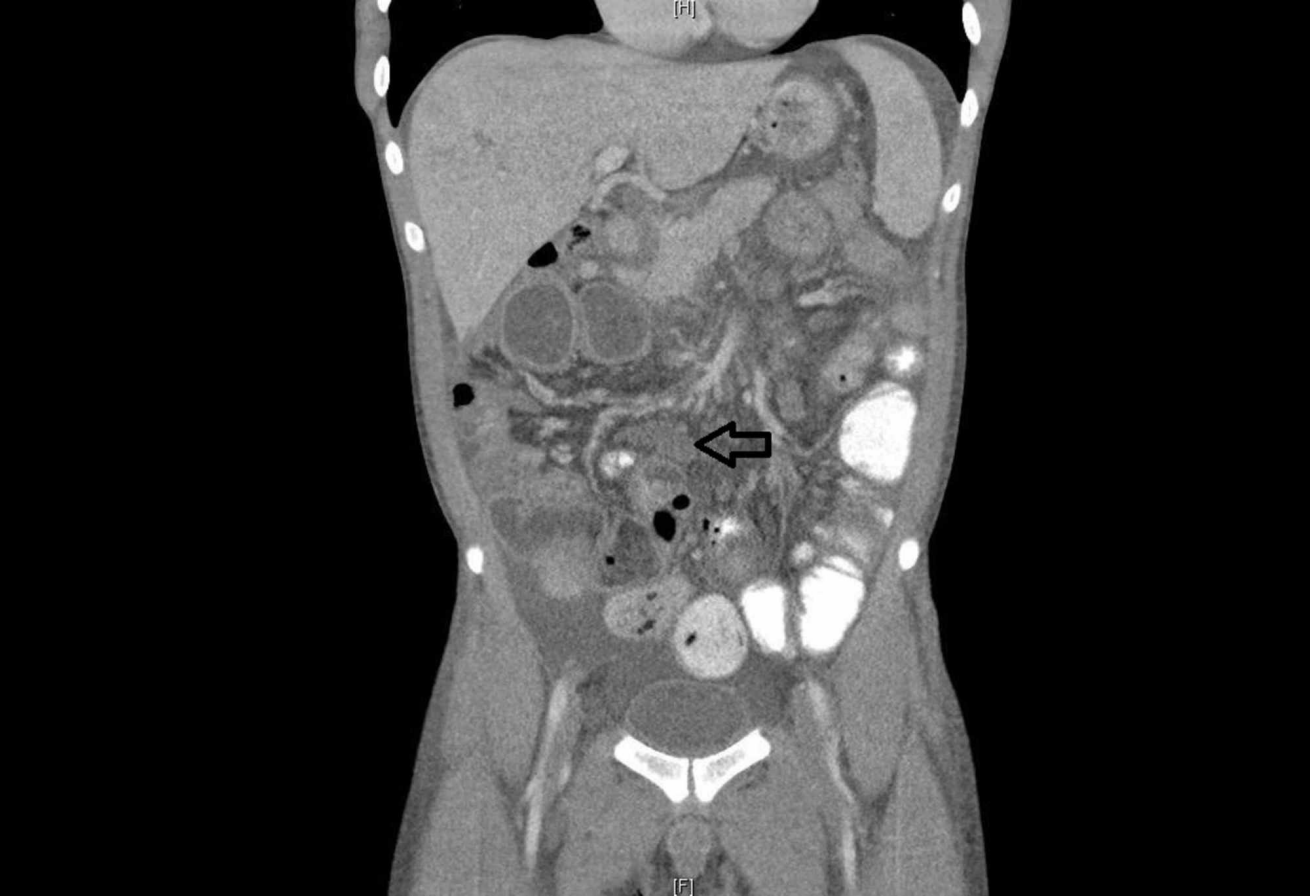
Sagittal section of CT abdomen/pelvis showing enlarged somewhat necrotic mesenteric lymph node (arrow).

The CT scan also showed multiple enlarged somewhat necrotic mesenteric lymph nodes. The figure shows a sagittal section of CT showing one of the mesenteric lymph node measuring 36 mm x 19 mm (Figure 2).

**Figure 3:**
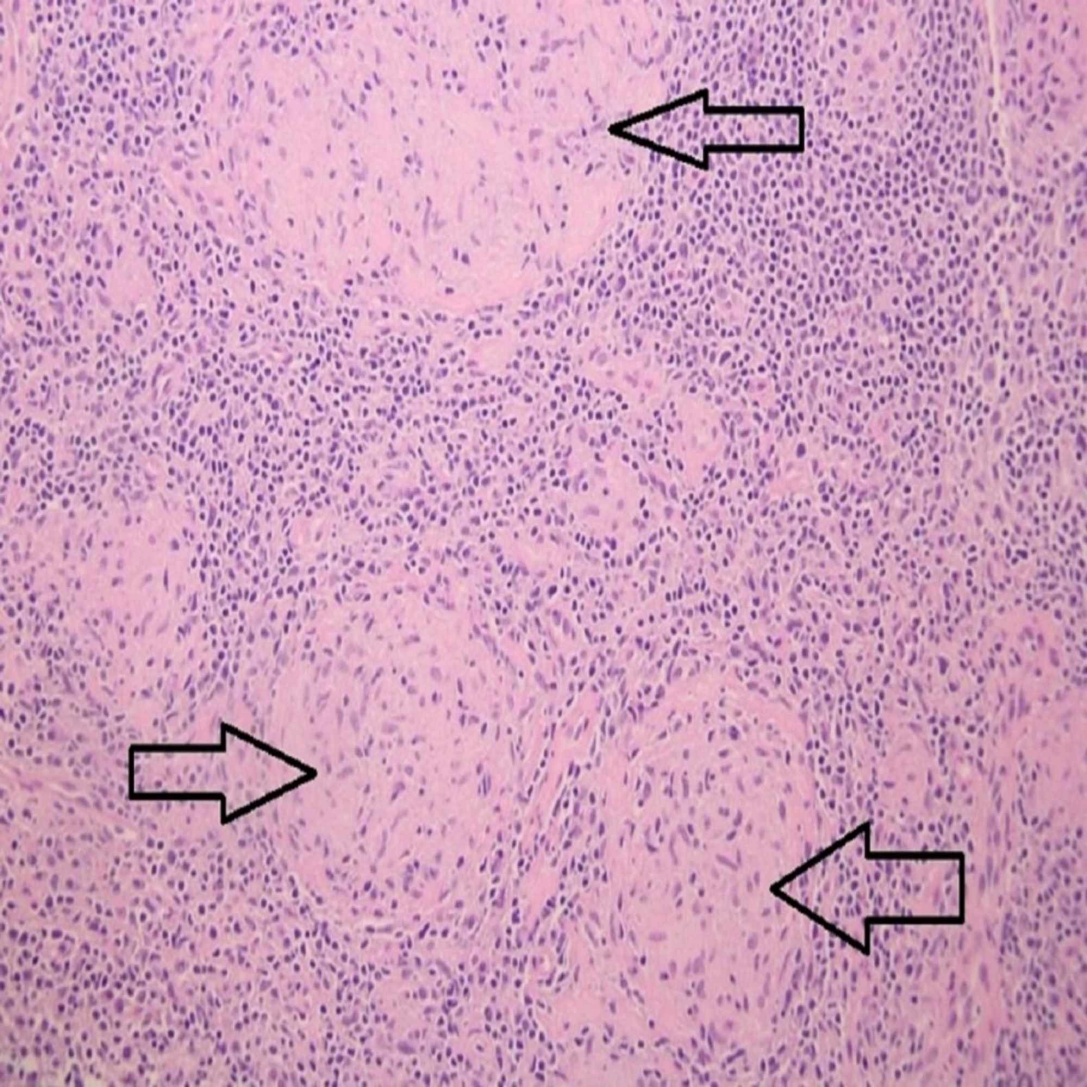
Mesenteric lymph node biopsy showing granulomas (arrows).

Gastric biopsy results showed acute and chronic inflammation with predominantly CD3 positive cells and were negative for malignancy or *H. pylori* staining. At this time, differential diagnosis was broad and included infection (bacterial, fungal, tubercular), autoimmune diseases (inflammatory bowel disease, Castleman’s disease, sarcoidosis), complement disorders like paroxysmal nocturnal hemoglobinuria, and malignancy including lymphoma. Lymph node and bone marrow biopsies were done to further evaluate his illness. His bone marrow biopsy was unremarkable and only showed reduced iron stores. Inguinal lymph node biopsy was inconclusive and showed fibrous tissue with scattered lymphoid aggregates. Mesenteric lymph node biopsy was done surgically and intraoperative findings showed several loops of dilated bowel with at least 10 counted areas of stricture highly concerning for CD. Lymph node biopsy results came back positive for noncaseating granulomas with negative infectious workup (Figure 3).

**Figure 4:**
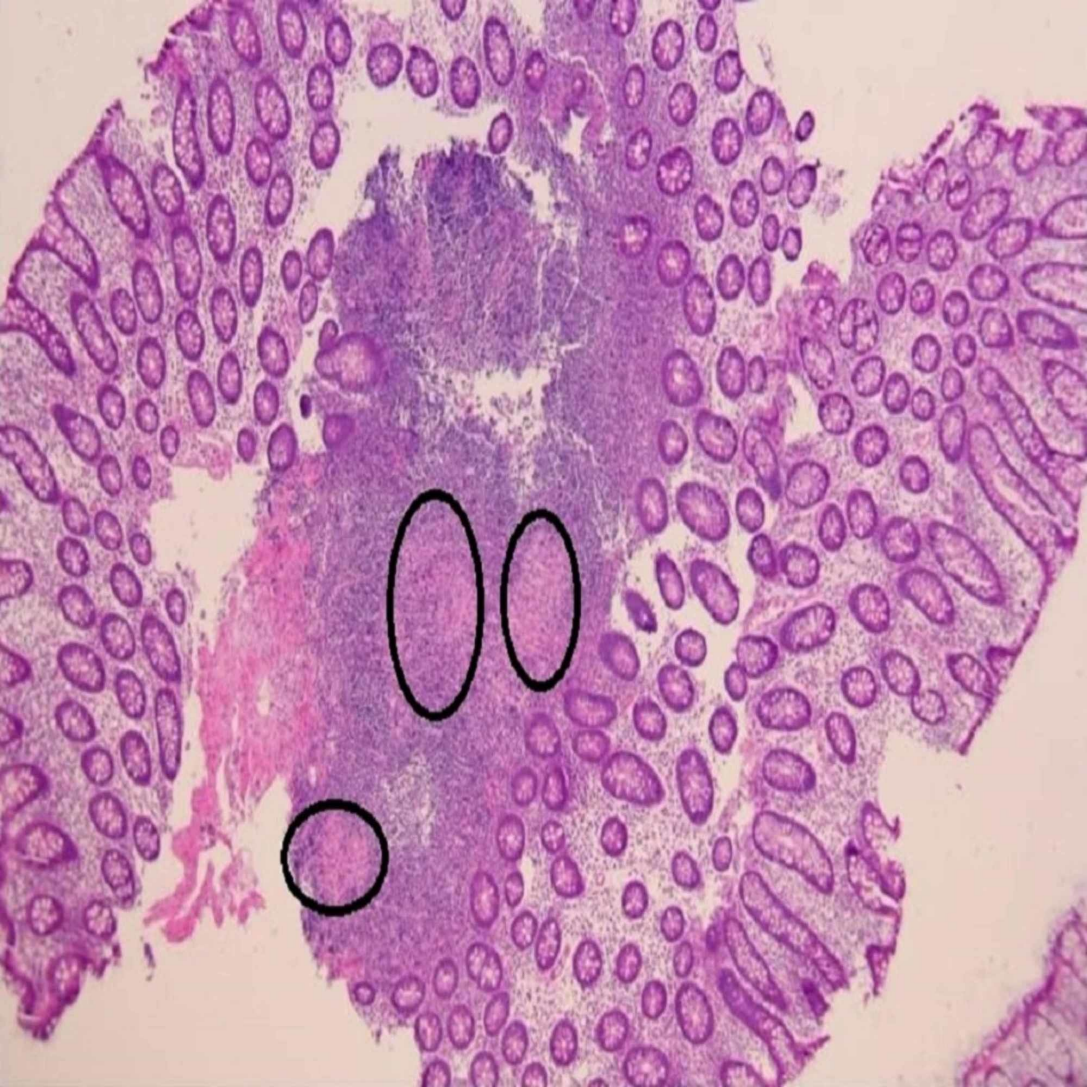
Normal colon with intact architecture. Circles demonstrate non-nectrotizing granulomas.

IgG and IL-6 levels were normal and his lymph node biopsy was not supportive of Castleman’s disease. Despite having extensive intra-abdominal disease his colonoscopy was grossly unremarkable, but the terminal ileum was unable to be visualized appropriately. Biopsies of the colon showed noncaseating granulomas (Figure 4).

The patient was subsequently discharged to continue outpatient follow-up for possible balloon enteroscopy. 

## Discussion

Venous thrombosis is a known and important complication of inflammatory bowel disease [5, 7]. The pathogenesis of thrombosis in IBD is complex and not fully explained. It is thought to be multifactorial as no consistent unifying etiology has been found yet. Many acquired factors may affect the hemostatic system and contribute to the pathophysiology of venous thromboembolism (VTE) in IBD patients. They include fluid depletion, prolonged immobilization, surgery, use of central venous catheters, steroid therapy, oral contraceptives or hormone replacement therapy, cigarette smoking, and vitamin deficiency leading to hyperhomocysteinemia. Many genetic mutations have been studied in IBD population but no significant difference has been found in their incidence in IBD patients when compared to healthy controls. Diffuse inflammation related to IBD is a pro-coagulable state that is known to cause both local as well as remote venous thrombosis [1-2, 8]. It can present as an incidental finding on imaging or can be the sole presentation of the disease [8]. Additionally, IBD-related venous thrombosis is being reported to present with variable severity [3, 6, 9]. The diagnosis of CD is usually established with endoscopic findings or imaging studies in a patient with a compatible clinical history. Colonoscopy is usually the first test of choice along with basic blood work. Different types of blood and fecal markers including anti-saccharomyces antibodies, p-ANCA, CRP, fecal calprotectin, and lactoferrin are used to help support the diagnosis [4]. Small bowel limited CD poses a challenge to diagnosis especially if the colonoscopy cannot be advanced into the terminal ileum. This patient had a fairly normal colonoscopic exam and the luminal part of his large intestine was devoid of any macroscopically appreciable disease. Characteristic findings of cobblestone appearance of the intestinal mucosa, transluminal inflammation, and ulcers were nowhere to be found on colonoscopy. Upper GI endoscopy was nondiagnostic as well. While capsule endoscopy comes in handy to diagnose such cases and can be utilized, it is not available widely even in many tertiary care hospitals [10-11]. This case helps remind us of the variable clinical presentation of CD and highlights the fact that despite the commonality of this disease, it still can be a diagnostic challenge.

## Conclusions

Crohn’s disease is an inflammatory and procoagulant state with a variable clinical presentation that requires a high index of suspicion for diagnosis. Venous thrombosis is an increasingly recognized presentation and complication of this disease. CD presenting with abdominal vein thrombosis is very rare and careful evaluation is required to rule out other diseases that can lead to a procoagulant state. Additionally, this case is extremely unique as we were unable to find significant intraluminal pathology on bidirectional endoscopies, which mostly is the characteristic of this disease. Presentation of CD with predominant extra-luminal inflammation with adhesions along with intra-abdominal venous thrombosis is very rare and poses a significant diagnostic challenge.
